# Anisotropic Strain Induced Directional Metallicity in Highly Epitaxial LaBaCo_2_O_5.5+δ_ Thin Films on (110) NdGaO_3_

**DOI:** 10.1038/srep37337

**Published:** 2016-11-21

**Authors:** Chunrui Ma, Dong Han, Ming Liu, Gregory Collins, Haibin Wang, Xing Xu, Yuan Lin, Jiechao Jiang, Shengbai Zhang, Chonglin Chen

**Affiliations:** 1 State Key Laboratory for Mechanical Behavior of Materials, Xi’an Jiaotong University, Shaanxi 710049, P. R. China; 2 Department of Physics and Astronomy, University of Texas at San Antonio, TX 78249, USA; 3 State Key Laboratory of Luminescence and Applications, Changchun Institute of Optics, Fine Mechanics and Physics, Chinese Academy of Sciences, Changchun 130033, P. R. China; 4 Department of Physics, Applied Physics, & Astronomy, Rensselaer Polytechnic Institute, Troy, NY 12180, USA; 5 Electronic Materials Research Laboratory and International Center for Dielectric Research, Xi’an Jiaotong University, Shaanxi 710049, P. R. China; 6 State Key Laboratory of Electronic Thin films and Integrated Devices, University of Electronic Science and Technology of China, Sichuan 610054, P. R. China; 7 Department of Materials Science and Engineering, University of Texas at Arlington, Arlington, Texas 76019, USA .

## Abstract

Highly directional-dependent metal-insulator transition is observed in epitaxial double perovskite LaBaCo_2_O_5.5+δ_ films. The film exhibit metallic along [100], but remain semiconducting along [010] under application of a magnetic field parallel to the surface of the film. The physical origin for the properties is identified as in-plane tensile strain arising from oxygen vacancies. First-principle calculations suggested the tensile strain drastically alters the band gap, and the vanishing gap opens up [100] conduction channels for Fermi-surface electrons. Our observation of strain-induced highly directional-dependent metal-insulator transition may open up new dimension for multifunctional devices.

With development of semiconductor technologies, devices with multifunctional properties are in increasing demand. Recently, many researchers are focus on the investigation of multifunction material by fabricating nanocomposite or multilayer thin film^1^,^2^. Not only nanocomposite or multilayer thin film with multifunctional properties but also a material with in-plane anisotropic properties is required for many applications^3^,^4^,^5^,^6^. The in-plane anisotropic resistivity induced by strain can be used to detect subtle changes in the external strain field from the environment^5^. Also, the in-plane anisotropic colossal magnetoresistance (CMR) has been demonstrated in epitaxial La_0.7_Sr_0.3_MnO_3_ thin film for the magnetic data storage^6^. Recently, the perovskite cobaltates have attracted increased attention due to their application as materials for oxidation catalyst, gas sensor, solid oxide fuel cell, and read/write heads in magnetic data storage^7^. In particular, LaBaCo_2_O_5.5+δ_ (LBCO) exhibits exotic electronic and magnetic properties from the intricate coupling of charge, spin, orbital, and lattice degrees of freedoms^8^,^9^,^10^. It has been reported that different the oxygen content in LBCO can lead to CoO_5_ pyramidal, CoO_6_ octahedral or mixed structure, and it can significantly influence its electric transport properties^11^,^12^. If there are the mixture of Co^3+^/Co^4+^ in the film, the film will shows the semiconductor or metallic behavior dependent on the test temperature range due to double exchange mechanism. When there is only Co^3+^ in the film, the film exhibits insulator behavior^11^. It is also found that with the increase of oxygen content in the LBCO thin film, the resistivity decrease at the low temperature^12^. Except the sensitivity to oxygen content, the physical properties of LBCO thin film are highly dependent on the type and amplitude of interface strain. It has been demonstrated that the isotropic interface strain induced by different cubic structure substrate improve colossal magnetoresistance of LBCO by 5 times of bulk material^13^, and the anisotropic interface strain (compressive strain along [100] and tensile strain along [010] relative to LBCO bulk material) induced by orthorhombic (110) NdGaO_3_ (NGO) substrate with the lattice parameters *a* = 5.433 Å, *b* = 5.503 Å, and *c* = 7.715 Å generate a stable and larger anisotropic resistivity in a wide temperature range from 300 K to 130 K^14^. Compared to the interface strain induced by different cubic substrate, the anisotropic interface strain generated by orthorhombic (110) NGO substrate with different in-plane lattice parameters (7.733 Å along the 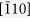
direction and 7.715 Å along [001] direction) can effectively exclude the influence of different growth modes and crystalline quality on different samples, since there is only variation for the sample on (110) NGO substrate. Thus, anisotropic interface strain generated by orthorhombic (110) NGO substrate is a very effective way to uncover the intrinsic nature of strain effects and the relaxation mechanism. However, the anisotropic properties of LBCO at low temperature (<130 K) can’t be investigated due to the resistance of LBCO is beyond the measurement limitation induced by the lower growth oxygen pressure and temperature.

Based on the previous study of LBCO thin film^14^, we plan to tune and optimize its properties by adjusting the growth oxygen pressure and temperature, which play a crucial role in determining the physical properties and structure of thin film. It is found that the increased growth oxygen pressure and temperature low down the resistivity of LBCO thin film, but the lattice constant of LBCO thin film is enlarged and an metal-insulator transition directionally occurs, namely, it only takes place in one of the in-plane directions, and in the other direction, the film maintains its semiconducting behavior. First-principle calculations indicate that the energy gap of the semiconducting LBCO decreases with tensile strain, and when the gap closes, electron conduction only takes place along one-dimensional channels. The unique directional metal-insulator transition of LBCO under anisotropic tensile strain with its clear physical understanding, we found for the first time, can be utilized for designing various novel devices, such as anisotropic magnetic data storage, simplified the integration of device, which needs metal in one direction and semiconductor in other direction, and so on.

## Results and Discussion

**Figure 1:**
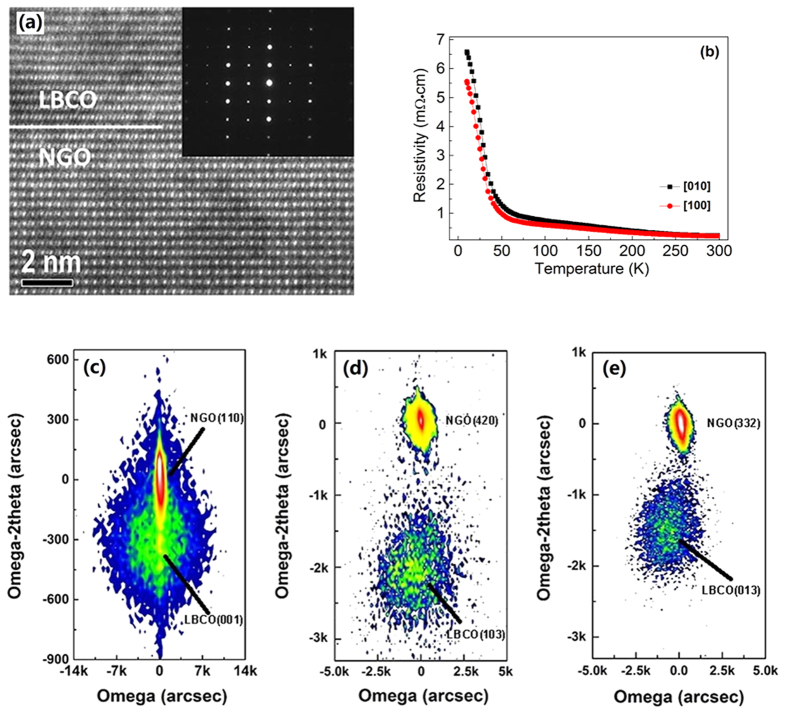
(**a**) High-resolution cross-sectional TEM image of LBCO thin films. The inset is the selected-area electron-diffraction patterns from an interface area. (**b**) Resistivity of the films along [100] and [010] change with temperature. Reciprocal-space maps around (**c**) LBCO (001) and NGO (110), (**d**) LBCO (103) and NGO (420), and (**e**) LBCO (013) and NGO (332).

From the high resolution x-ray diffraction spectra, it is found that only (00 *l*) peaks appear in the *θ*-2*θ* scans, suggesting that the as-grown films are *c*-axis oriented. The films exhibit excellent epitaxial quality with atomically-sharp interfaces, as revealed by the high-resolution cross-sectional transmission electron microscope (TEM) image in Fig. 1(a). Inset in Fig. 1(a) shows the selected-area electron-diffraction (SAED) patterns from an interface area that covers both the substrate and thin film. The sharp electron diffraction spots suggest that the as-grown LBCO thin films have good single crystallinity, as no evidence of satellites or broadening can be seen. The resistivity of LBCO thin film along the [100] and [010] in-plane directions were measured by using the Physical Property Measurement System (PPMS)-9. From the Fig. 1(b), it is clearly see that the resistivity of LBCO is lowered down by adjusting the growth oxygen pressure, but the resistivity of [100] direction is smaller than that of [010] direction, which is opposite to the previous report^14^. In order to understand the underlying mechanism, reciprocal-space maps (RSMs) are recorded around the (001), (013), and (103) reflections of the LBCO films (Fig. 1(c)) to get a clear picture on the out-of-plane and in-plane lattice constants. The reflection spot from the film overlaps with that from the substrate and no measurable Δ*ω* can be discerned between the LBCO (001) peak and NGO (110) peak. These suggest that the (001) plane of the LBCO film is parallel to the (110) plane of the NGO substrate without any detectable tilt. To be more certain on this result, Fig. 1(d) shows the RSMs around the asymmetric reflections of LBCO (103) and NGO (420), acquired using a glancing exit scan, and Fig. 1(e) shows the RSMs around the asymmetric reflections of LBCO (013) and NGO (332) with the same experimental setting but a 90° rotation of *ϕ*. It is found that the epitaxial relationship is (001)_LBCO_//(110)_NGO_ (out-of-plane), [100]_LBCO_//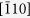
_NGO_ and [010] _LBCO_//[001]_NGO_ (in-plane), and the in-plane relationship is opposite to the case of the lower growth oxygen pressure and temperature ([100] _LBCO_//[001]_NGO_ and [010]_LBCO_//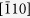
_NGO_)^14^. From Bragg law and the angular relationship between these crystalline planes^15^, the lattice parameters of the LBCO thin films are calculated to be *a* = 3.995 Å, *b* = 3.939 Å, and *c* = 3.845 Å, which is totally different from the case of lower growth oxygen pressure and temperature (*a* = 3.86 Å, *b* = 3.90 Å, and *c* = 3.97 Å), indicating that the growth oxygen pressure and temperature is a very key factor to determine the growth of the LBCO thin film and its the lattice constant. It is very strange that the in-plane lattice parameters *a* and *b* are larger than that of ordered La_0.5_Ba_0.5_CoO_3_ bulk (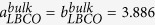 Å)^8^, since 
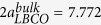
 Å > 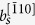 = 7.733 Å and 2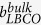 = 7.772 Å > 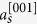 = 7.715 Å, the substrate is thus *expected* to cause in-plane compressive strains, as well as an out-of-plane tensile strain on the epitaxial LBCO film. Actually, however, the RSM results show in-plane tensile strains and out-of-plane compressive strain for the epitaxial film, which are completely opposite to the predictions by the above simple calculation. To understand the discrepancy, we notice that the thermal expansion coefficients of cubic LBCO (*α*_*LBCO*_ = 23.47 × 10^−6^/°C)^16^ and NGO (*α*_*NGO*_ = 10 × 10^−6^/°C)^17^ are noticeably different. At the growth temperature (850 °C), the LBCO film is nearly cubic with a bulk lattice parameter of 3.961 Å, namely, its composition is almost that of LaBaCo_2_O_6_. As such, the lattice parameters nearly match with those of the substrate. Indeed, (7.922–7.780)/7.780 = 1.8% in 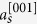 and (7.922–7.797) = 1.6% in 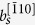 are both reasonably small. When the samples are annealed to room temperature or below at which electrical measurements were taken, due to the epitaxy, the change in the lattice parameters of the thin film follows the thermal expansion of the NGO substrate, not that of LBCO. If the tetragonal symmetry of LaBaCo_2_O_6_ were maintained, the in-plane lattice parameters of the LBCO should be *a* = *b* = 3.929 Å. This is consistent with the RSM result of *b* = 3.939 Å along [010] for LBCO, but is not consistent with the result of *a* = 3.995 Å along [100]. Given that the strain effect during the growth is relatively small, a larger lattice parameter for *a* ([100]) suggests that the cooling process is accompanied by something else, most likely, by the formation of oxygen vacancies along [100], since the repulsive force between cations will enlarge the lattice constant as a result of the missing of oxygen between cations^12^. The formation of oxygen vacancies probably results from the increase of the growth temperature, since the amount of vacancies is proportional to the temperature in spite of the increase of growth oxygen pressure^18^. The ordered oxygen vacancies in nanoscale already be detected in LBCO thin film^19^, but it is impossible to accurately figure out the amount of oxygen vacancies in LBCO thin films due to the Co L3/L2 intensity ratios between stoichiometric and non-stoichiometric layers in the perovskite structure do not show any appreciable changes from the electron energy loss spectroscopy (EELS), thus the valence state(s) of the Co cannot be identified from EELS^20^,^21^. Also, one cannot use the oxygen K edge intensity to estimate the local stoichiometry of the O-depleted layers, because with the decrease of the annular dark- field intensity, the EELS intensity also increases. In short, an anistropic in-plane tensile strain is generated in the LBCO thin film, and oxygen vacancies induce larger tensile strain along [100] direction.

**Figure 2:**
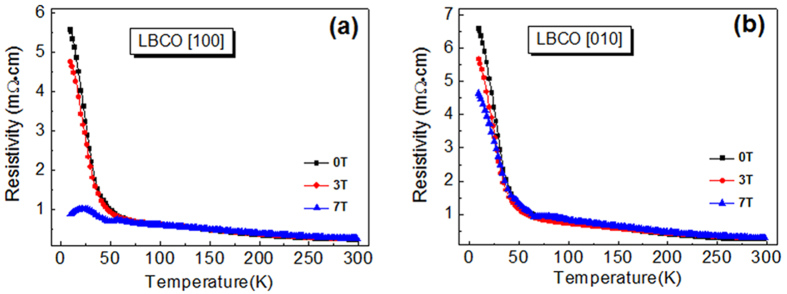
Resistivity of the films along (**a**) [100] and (**b**) [010] as a function of temperature under different magnetic field.

Besides the interesting results in the microstructure, a most striking result is found in the electrical transport measurement. As shown in Fig. 2, when a magnetic field of 7 T is applied parallel to the surface of the thin films, a metal-insulator transition (~25 K, Fig. 2(a)) takes place along [100], the film is still semiconducting along [010] (Fig. 2(b)). Moreover, it is clearly seen that there is a change of slope at around 50 K, which is probably related to the transition of ferromagnetic (FM) and antiferromagnetic (AFM) of LBCO thin film^13^.

In order to understand the experimental findings, first-principle calculations, based on the density-functional theory (DFT), were carried out by using the VASP code^22^. The detail calculation information are shown in the method part. From X-ray data and the properties of LBCO thin film, our oxygen content in this LBCO film is somewhere 0 < δ < 0.5. To develop a qualitatively understanding, here we consider δ = 0, namely LaBaCo_2_O_5.5_, which is computationally manageable with small enough unit cell and more consistent with the experiment data. The LaBaCo_2_O_5.5_ model contains 38 atoms built from a 2 × 2 × 1 LaBaCo_2_O_6_ supercell by removing two oxygen atoms from the La layer according to the report by Rautama *et al.*^10^ and the observation of nanoscale ordered oxygen vacancies in LBCO thin film^19^.

**Figure 3:**
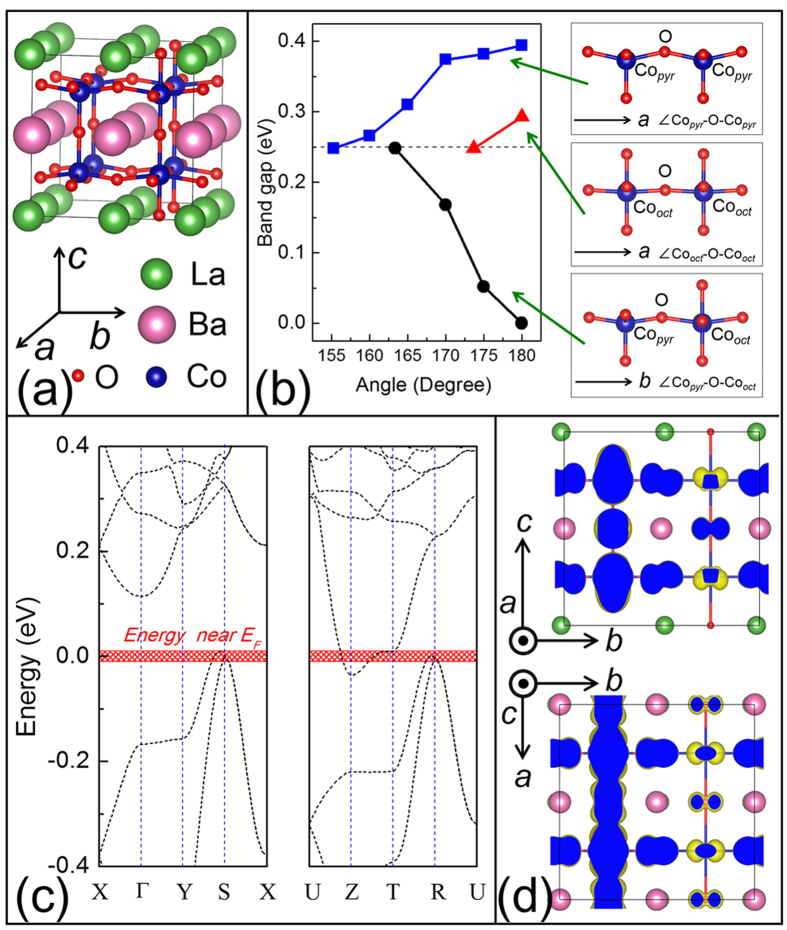
(**a**) Optimized geometry of LaBaCo_2_O_5.5_ in the AF-phase (G-type). (**b**) Band gap change with respect to the change of the Co-O-Co angles. These angle changes are from those of the optimized geometry to the tensile-stained geometry with Co-O-Co angles = 180°. (**c**) Band structure of the strained LaBaCo_2_O_5.5_, with energy near the Fermi level (E_F_ ± 0.015 eV) marked and (**d**) the corresponding charge distribution in the real space with an isosurface (yellow color) of 2 × 10^−4^ *e*/Å^−3^. Blue regions are cuts through the isosurfaces.

Figure 3(a) shows the optimized low-energy structure for LaBaCo_2_O_5.5_, and it can be seen that the oxygen vacancies prefer to form directional chains along *a* direction ([100]), resulting in an expansion of lattice parameters *a* and *b*, and *a* > *b*, which agrees with the x-ray measurement. Table 1 (second row) shows the calculated lattice parameters, which are in good agreement with the experimental result (given in the first row). Here, a larger-than-experiment *a* is consistent with the fact that in the experiment δ > 0 and the metal-insulator transition occur at low temperature not room temperature.

Stable LaBaCo_2_O_5.5_ is AFM, which is 26 meV/atom lower in energy than the FM phase. This energy is comparable with the thermal energy at room temperature (*k*T = 26 meV). The AFM phase is semiconducting with a band gap of 0.25 eV, whereas the FM phase is metallic. Hence, at a reasonable temperature, the AFM phase should be the majority phase, whereas the FM phase could be a minority phase. Note that the actual LBCO thin film is δ > 0, and tensile strained in in-plane direction. It is known that often a small change (bond angle and bond length) in the peroverskite oxides can significantly change its physical properties as a result of the strong electron-lattice coupling, and the bond angle is easier to be changed than the bond length by the outside environment^23^. This raises the question whether the unexpected behavior of the LBCO thin film (δ > 0) is a manifestation of the anisotropic in-plane tensile strain effect. To mimic the tensile strain effects at low temperature, we calculate the band gap change with respect to the bond angles from that of the optimized geometry, since the bond angle of LBCO is derived from the ideal structure 180° 9. From Fig. 3(a), it is clearly seen that the LaBaCo_2_O_5.5_ consists of two in-equivalent cobalt sites–the octahedral and pyramidal sites, out of which there exist three different Co-O-Co bond angles: namely, Co_*pyr*_-O-Co_*pyr*_ (along [100] direction) Co_*oct*_-O-Co_*oct*_ (along [100] direction) and Co_*pyr*_-O-Co_*oct*_ (along [010] direction). Figure 3(b) shows the band gap change with these three type bond angle. It is found that only stretched Co_*oct*_-O-Co_*pyr*_ ([010] direction) lowers the band gap considerably from 0.25 eV to zero gap when the angle is around 180°, indicating that it can aid to lower the resistivity of LBCO thin film and increase the conductivity. Thus, the combination of the tensile strain along [010] direction and oxygen vacancies can lower the band gap of LBCO thin film. Figure 3(c) shows the band structure when the bond angle Co_*oct*_-O-Co_*pyr*_ equals to 180°. From Fig. 3(c), it can be seen that the Fermi level passes through the valence band at the S and R points of the Brillouin zone and the conduction band at the Z point. Since electrical transport only involves states near the Fermi level (*E*_*F*_), we show the real-space carrier distribution in Fig. 3(d) over an energy range of ±15 meV from the *E*_*F*_, from which we see that the distribution is highly asymmetrical: states along the Co_*pyr*_-O-Co_*pyr*_ chains in the *a* ([100]) direction are connected, whereas those in the other directions (*b* and *c*) are not. Electrons injected from electrode can be viewed as a wave packet, whose transport requires the coupling to available states near *E*_*F*_ in the direction of the transport. Hence, Fig. 3(d) suggests that, under such a condition, electron transport primarily takes place in the large-tensile-strain *a* ([100]) direction. A qualitative picture thus emerges that may help us understand the experiment: (i) The LBCO thin film suffers anistropic in-plane tensile strain, and the larger tensile strain induced by the oxygen vacancies is along *a* ([100]) direction. (ii) With temperature decrease, the films will suffer an even stronger tensile strain from the substrate, and its band gap will decrease by increasing the angle of Co_*oct*_-O-Co_*pyr*_ in the *b* ([010]) direction. And (iii) when the band gap is closed, a magnetic field may be required to generate the metallic transport in *a* ([100]) direction, since defects and domain boundaries, which actually exists in the film and acts as energy barrier, are not taken into account in the calculation. The magnetic field will promote electron spin to align along the direction of the field and reduce carrier scattering, resulting in the decrease of resistivity and the occurrence of insulator-metal transition along [100] direction.

## Conclusions

In summary, a directional metal-insulator transition behavior of the LBCO thin film on (110) NGO substrate was observed, indicating that the anisotropic in-plane strains lead to new physical properties. With the aid of magnetic field, the metal-insulator transition takes place only along [100], but the film maintains its semiconducting behavior along [010]. First-principles calculations give a good explanation and suggest that under the condition of that tensile strain of [010] direction close the band gap, a conduction channel along [100] will open for electron transport, generating the metal-insulator transition at low temperature along [100]. These results not only deepen the understanding of stain-dependent physical properties in LBCO films, but also demonstrate the feasibility of achieving the coexistence of metal and semiconducting behaviors in one material by simply applying anisotropic strains.

## Methods

A batch of LBCO thin films were fabricated on (110) NGO substrate by pulsed laser deposition using a KrF excimer laser with a wavelength of 248 nm. A laser energy of 2.0 J/cm^2^ was selected, and a deposition temperature increase from 800 °C to 850 °C as well as the growth oxygen pressure increase from previous reported 20 mTorr to 350 mTorr^14^. After the deposition, the LBCO films were annealed *in situ* at 850 °C for 15 mins in a pure oxygen atmosphere at 200 Torr and then slowly cooled down to room temperature with a rate of 5 °C/min.

First-principles calculations based on the density-functional theory (DFT) were carried out using the VASP code^22^. Projector augmented wave basis^24^ and Perdew-Burke Ernzerhof functional^25^ were employed. The cutoff energy for plane-wave basis and Monkhorst-Pack *k*-point mesh grid were set to 600 eV and 3 × 3 × 3, respectively. More accurate calculations by using 5 × 5 × 5 k-point mesh grid showed the same band structure and the total energy difference was smaller than 0.05 eV. The LaBaCo_2_O_5.5_ model contains 38 atoms, which is built from a 2 × 2 × 1 LaBaCo_2_O_6_ supercell by removing two oxygen atoms from the La layer. The structure agrees with experiment^10^. During the spin-polarized calculation, all of the atoms were relaxed until the Hellman-Feynman force is less than 0.014 eV/Å. For cobalt, we used the GGA+U method for Co *3d* orbital, with the Coulomb interaction *U* = 5 eV and exchange interaction *J* = 0.9 eV. Changing the U by Δ*U* = ±1 eV has negligible effect on the results, as having been demonstrated by others before^26^,^27^.

## Additional Information

**How to cite this article**: Ma, C. *et al.* Anisotropic Strain Induced Directional Metallicity in Highly Epitaxial LaBaCo_2_O_5.5+δ_ Thin Films on (110) NdGaO_3_. *Sci. Rep.* **6**, 37337; doi: 10.1038/srep37337 (2016).

**Publisher’s note:** Springer Nature remains neutral with regard to jurisdictional claims in published maps and institutional affiliations.

## Figures and Tables

**Table 1:** The percentage change of the lattice parameters of LaBaCo_2_O_5.5+δ_ thin films, compared to bulk.

Lattice Parameters	*a* = [100]	*b* = [010]	*c* = [001]
Experiment	+2.8%	+1.4%	−1.0%
Calculation	+4.9%	+1.4%	−1.1%

Positive (negative) sign represents an increase (decrease).
